# Vulnerability Index Approach to Identify Pharmacy Deserts and Keystone Pharmacies

**DOI:** 10.1001/jamanetworkopen.2025.0715

**Published:** 2025-03-13

**Authors:** Walter S. Mathis, Lucas A. Berenbrok, Peter A. Kahn, Giovanni Appolon, Shangbin Tang, Inmaculada Hernandez

**Affiliations:** 1 Department of Psychiatry, Yale School of Medicine, New Haven, Connecticut; 2 Department of Pharmacy and Therapeutics, University of Pittsburgh School of Pharmacy, Pittsburgh, Pennsylvania; 3 Section of Pulmonary, Critical Care, and Sleep Medicine, Yale School of Medicine, New Haven, Connecticut; 4 Herbert Wertheim School of Public Health and Human Longevity Science, University of California, San Diego, La Jolla; 5 Division of Clinical Pharmacy, Skaggs School of Pharmacy and Pharmaceutical Sciences, University of California, San Diego, La Jolla

## Abstract

**Importance:**

Community pharmacies are crucial for public health, providing essential services such as medication dispensing, vaccinations, and point-of-care testing. Addressing disparities in pharmacy access, particularly in underserved rural and low-income areas, is critical for health equity.

**Objective:**

To identify areas in the US at risk of becoming pharmacy deserts through the development of a novel pharmacy vulnerability index.

**Design, Setting, and Participants:**

This population-based cross-sectional study in the contiguous 48 states performed geographic information systems analysis of pharmacy data from the National Council for Prescription Drug Programs (NCPDP) dataQ. Participants included all open-door pharmacies (community or retail pharmacies open to the general public without restrictions on who can access its services) in the US as of February 2024. Statistical analysis was performed from July to August 2024.

**Exposure:**

The primary exposure was travel time to pharmacies across the US.

**Main Outcomes and Measures:**

A pharmacy desert was defined as a census tract where the travel time to the nearest pharmacy exceeds the supermarket access time for that region and urbanicity level. Building on this definition, a pharmacy vulnerability index was developed, which indicates the number of pharmacies that would need to close for a census tract to become a pharmacy desert. Tracts with a pharmacy vulnerability index of 1, depending solely on a single pharmacy for access, were identified as at risk of becoming deserts. Subpopulation totals and percentages living in pharmacy deserts or relying on keystone pharmacies were computed, and then stratified by urbanicity and race.

**Results:**

Among 321.3 million individuals (39.7 million [12.3%] Black, 59.0 million [18.2%] Hispanic, 195.0 million [60.3%] White) in the contiguous US, 57.1 million (17.7%) were identified as living in pharmacy deserts, with 28.9 million (8.9%) additionally relying on a single pharmacy for access. Small rural areas were particularly affected, with a higher dependency on single pharmacies (4.1 million individuals [14.3%]).

**Conclusions and Relevance:**

In this cross-sectional study of pharmacy access in the US, significant disparities in pharmacy access were identified, especially pronounced in small rural areas. Targeted policy interventions, such as incremental reimbursement rates or other monetary incentives, are needed to ensure the financial sustainability of pharmacies that serve as the sole source of pharmacy services in at-risk areas.

## Key Points

**Question:**

Can areas in the US at risk of becoming pharmacy deserts be better identified by developing a pharmacy vulnerability index?

**Findings:**

This cross-sectional study of the US population (321.3 million people) found that 17.7% resided in pharmacy deserts, and an additional 8.9% relied on a single pharmacy for access. Notably, individuals in small rural areas were particularly at risk for pharmacy access issues.

**Meaning:**

These results suggest that targeted policy interventions are essential to prevent further inequities in pharmacy access, particularly in small rural areas, by ensuring the financial sustainability of pharmacies that are the sole source of pharmacy services in an area.

## Teaser

This cross-sectional study uses a pharmacy vulnerability index to identify areas in the US at elevated risk of becoming pharmacy deserts.

## Introduction

Access to community pharmacies is essential for ensuring medication access and promoting public health. More than two-thirds of US adults use at least one medication, and over 90% of prescriptions are filled at community pharmacies.^1,2^ Beyond dispensing medications, community pharmacies play a substantial role in administering vaccines, providing rapid diagnostics,^3^ and offering medication management services.^4,5^ Community pharmacies that directly serve the general public are crucial for health care equity, serving individuals who lack access to other health care settings.^4^ The recent trend of pharmacy closures, particularly among independent pharmacies in low-income urban and rural areas, threatens equitable access to care.^6^

Research on pharmacy access has primarily focused on identifying *pharmacy deserts*, a concept adapted from food deserts. While there are many working definitions of pharmacy desert,^7^ the one most frequently used refers to census tracts with a high poverty rate and no pharmacies within 1 mile in urban areas or 10 miles in rural areas.^8^ Some efforts have updated this definition with travel time thresholds.^7,9^ However, the pharmacy desert framework has substantial limitations. First, distance and/or time thresholds are often arbitrarily set based on convenience (eg, 1 mile or 15 minutes) rather than empirical evidence. Second, nationwide definitions overlook important geographic variability in normative travel times or distances across the country. Third, the binary classification of a pharmacy desert fails to assess the risk level of a community’s pharmacy access, as areas relying on a single pharmacy for access are categorized the same as those with multiple pharmacies. Additionally, including a high poverty rate as a criterion, borrowed from the food desert definition, was intended to exclude areas where individuals had the means to bypass access barriers, but failed to consider that individuals most at risk for access challenges live in both high- and low-income areas.^10^

Beyond the methodological limitations in defining pharmacy deserts, previous research on pharmacy access has been hindered by the limited policy levers available to influence the establishment of new pharmacies. This challenge is partly due to the substantial investment required to open a pharmacy.^11^ In response, we propose identifying census tracts at risk of becoming pharmacy deserts as a focus for immediate policy intervention.

With this objective in mind, we propose a redesign of the research framework to assess spatial access to health care infrastructure. We introduce a refined definition of a pharmacy desert based on travel time rather than distance, which is responsive to regional characteristics and independent of income. Specifically, a pharmacy desert is defined as a census tract where the travel time to the nearest pharmacy exceeds the supermarket access time threshold for that region and urbanicity level. We select supermarkets to derive access time thresholds because they are among the most common retail outlets and a regular destination for most households,^12,13^ and they have accessibility patterns that reflect region-specific travel norms. These patterns are not random but are shaped by underlying factors such as market forces, population densities, land use patterns, and consumer travel behaviors, which vary substantially by region and level of urbanization.^14^

Building on this definition, we introduce the pharmacy vulnerability index—a metric that indicates the number of pharmacies that would need to close for a census tract to become a pharmacy desert. Tracts with a pharmacy vulnerability index of 1, depending solely on a single pharmacy for access, are identified as at risk of becoming deserts. We also propose the term *keystone pharmacies* for these critical locations whose closure would directly create a pharmacy desert. Although tailored for pharmacy access, our approach can be adapted to analyze nearly any essential service where physical access is crucial, whether in health care, such as hospitals and emergency departments, or in other sectors.

## Methods

### Pharmacy Data

We obtained from the National Council for Prescription Drug Programs (NCPDP) 2024 dataQ, which contains information for all pharmacy locations across the US, including addresses.^15^ We selected pharmacy locations open as of February 2024 and constrained sampling to open-door pharmacies, including retail pharmacies and Indian Health Service pharmacies. Institutional, mail-order, and government pharmacies were excluded from analyses as they are generally not open for external walk-in patients. We geocoded pharmacy locations in ArcGIS Pro 3.2.2 software with ArcGIS StreetMap Premium.^16,17^ Travel time computation was performed using an Open Source Routing Machine instance operating on OpenStreetMap data.^18,19^ The study did not constitute human subjects research as only pharmacy location and deidentified census data were used, so it did not require institutional board review or informed consent, in accordance with 45 CFR §46. This study followed the reporting requirements of the Strengthening the Reporting of Observational Studies in Epidemiology (STROBE) reporting guideline for cross-sectional studies.^20^

### Deriving Thresholds for the Definition of Deserts

We adopted empirically derived, region- and urbanicity-normed drive time thresholds from our previous study on supermarket access to define pharmacy deserts.^21^ Supermarkets are among the most ubiquitous and universally needed retail resources^22^ and have been foundational in pharmacy and food desert analysis.^8,23^ Using the same datasets (TDLinx and SNAP) and supermarket definitions as the USDA did when defining food deserts,^24^ we applied supermarket-derived thresholds to measure pharmacy access. Briefly, these thresholds were derived by determining for each commuting region and urbanicity the minimum drive time from a supermarket within which 80% of the population lives (eFigure and eTable 1 in Supplement 1).

Urbanicity was defined using a 4-category (urban core, suburban, large rural, and small town/rural) aggregation of the rural-urban community area (RUCA) census tract designations (eTable 2 in Supplement 1).^25,26^ Regions were adopted from a sophisticated study of US commuting patterns to define areas within which people tend to commute,^27^ and hence would likely have similar drive time norms. Analysis was limited to the contiguous 48 states plus the District of Columbia. Race and ethnicity data used in this study were extracted from the US Census American Community Survey 2020 5-year estimates, where data were collected via self-report using categories defined by the Office of Management and Budget, including but not limited to American Indian or Alaska Native, Asian, Black or African American, Hispanic or Latino, Native Hawaiian or Other Pacific Islander, and White.^28^ We used 3 racial categories (Black, Hispanic, White) to align with over a decade of pharmacy access research, including studies by Qato et al^8,27^ and our own 2022 work,^8^ ensuring methodological continuity, facilitating comparisons between methods, and capturing the largest minoritized groups in the US.

### Statistical Analysis

We defined pharmacy vulnerability index as the number of pharmacies that would need to close for a census tract to be considered a desert. A vulnerability index score of 1 designates communities relying on a single pharmacy for access. We refer to these pharmacies which are the only source of pharmacy services in an area, such that their closure would result in a pharmacy desert as *keystone pharmacies*.

Pharmacy vulnerability indices were computed for each census tract as follows: First, for all census blocks within each census tract, we identified the 25 pharmacies closest to the block centers in terms of straight-line distance. Then, we computed driving times between all blocks and each of these pharmacies, recording for each block the minimum drive time and the pharmacy. We aggregated census block level data at the census tract level by averaging the minimum drive times for each block in the tract weighted by its population. We define a pharmacy desert as a census tract where the population-weighted mean travel time to a pharmacy was greater than the supermarket access threshold for that region and urbanicity. We then computed the vulnerability index for each census tract by determining the minimum number of pharmacies that would need to be removed to make the tract a pharmacy desert by iteratively removing the remaining pharmacy with the lowest population-weighted mean minimum travel time and determining desert status.

**Figure 1.**
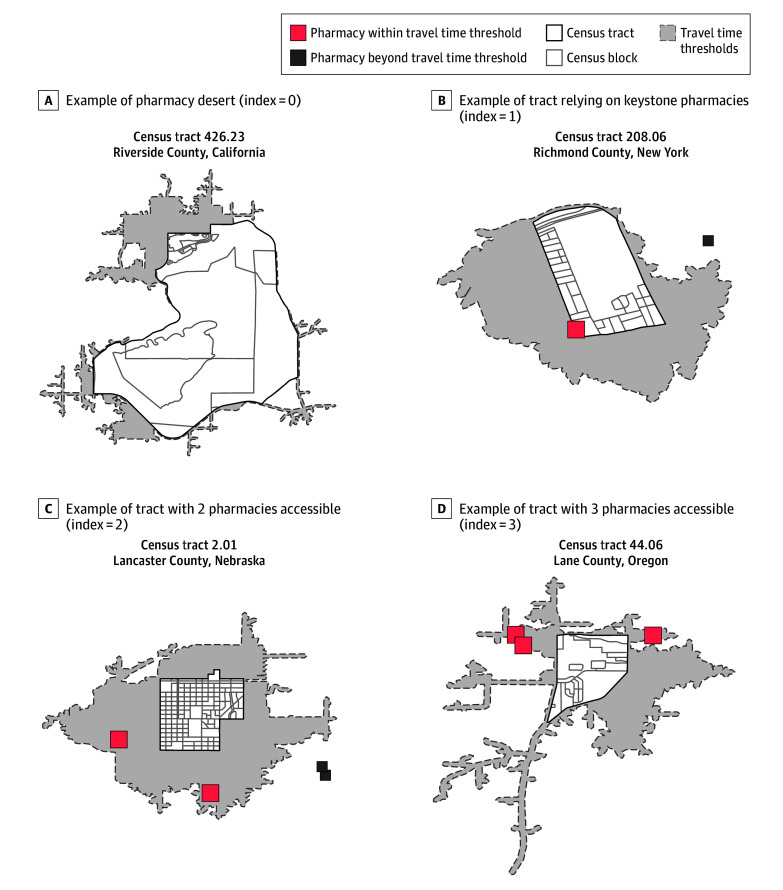
Examples of Pharmacy Vulnerability Indices

Figure 1 exemplifies this categorization. Census tract 426.23 in Riverside County, California, is a pharmacy desert as there was no pharmacy within the travel time threshold based on the commuting region and level of urbanicity. Census tract 208.06 in Richmond County, New York, relies on a keystone pharmacy for access, as there was only 1 pharmacy within the travel time threshold. Census tracts 2.01 in Lancaster County, Nebraska, and 44.06 in Lane County, Oregon, were examples of census tracts where multiple pharmacies could be removed before becoming a pharmacy desert.

To facilitate interpretability and statistical analysis, tracts were divided into discrete categories by their vulnerability index scores. Given their specific, interpretable meaning, values of 0 and 1 were given their own categories. Two other break points were determined such that all other tracts were divided into 3 categories with counts as close to equal as possible. We calculated the subpopulations living in each of these 5 categories, stratified by urbanicity and race. Statistical analyses were performed using R statistical software version 4.3.3 (R Project for Statistical Computing) from July to August 2024.

## Results

**Figure 2.**
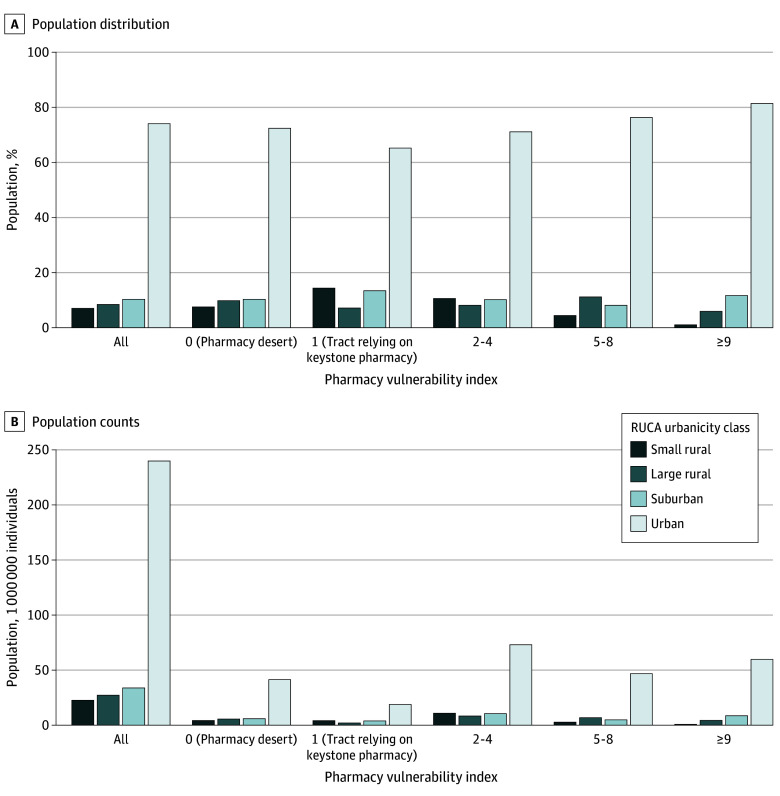
Distribution of Population by Pharmacy Vulnerability Index Across Urbanicity Groups Pharmacy desert was defined as a census tract lacking a pharmacy within the established drive time threshold, based on the region and level of urbanicity. Keystone pharmacy was defined as a critical pharmacy whose removal would result in the conversion of a census tract into a pharmacy desert. RUCA indicates rural-urban community area.

Among the 323.5 million individuals (39.7 million [12.3%] Black, 59.0 million [18.2%] Hispanic, 195.0 million [60.3%] White) in the contiguous US at the time of this study, 57.1 million (17.7%) lived in pharmacy desert census tracts, and 28.9 million (8.92%) lived in tracts that rely on 1 of 5610 keystone pharmacies (Table 1, Figure 2; eTable 3 in Supplement 1). Although independent or franchise pharmacies represented 24 288 of 61 046 pharmacies across the US (39.8%), they represented 2519 of 5610 keystone pharmacies (44.9%) (eTable 3 in Supplement 1). Chain pharmacies represented 36 456 of 61 046 pharmacies across the US (59.7%), but only 3047 of 5610 keystone pharmacies (54.3%).

**Table 1.** Population Totals by RUCA Class and Pharmacy Vulnerability Index^a^

RUCA class	Pharmacy Vulnerability Index					
	0 (n = 57 134 374)	1 (n = 28 856 229)	2-4 (n = 102 816 187)	5-8 (n = 61 250 932)	≥9 (n = 73 430 899)	Total (N = 323 488 621)
Small rural	4 263 205 (7.5)	4 126 770 (14.3)	10 895 603 (10.6)	2 676 316 (4.4)	713 638 (1.0)	22 675 532 (7.0)
Large rural	5 615 026 (9.8)	2 052 519 (7.1)	8 369 846 (8.1)	6 850 768 (11.2)	4 318 551 (5.9)	27 206 710 (8.4)
Suburban	5 876 415 (10.3)	3 862 985 (13.4)	10 499 548 (10.2)	4 986 060 (8.1)	8 622 736 (11.7)	33 847 744 (10.5)
Urban	41 379 728 (72.4)	18 813 955 (65.2)	73 051 190 (71.1)	46 737 788 (76.3)	59 775 974 (81.4)	239 758 635 (74.1)

Abbreviation: RUCA, rural-urban commuting area.

^a^ The pharmacy vulnerability index represents the number of pharmacies that would need to close so that a census tract would become a pharmacy desert. Tracts with a vulnerability index of 0 represent pharmacy deserts; tracts with a vulnerability index of 1 are considered at risk of becoming deserts due to their reliance on a single location for access.

### By Urbanicity

Among 57.1 million individuals who lived in pharmacy deserts across the US, 41.4 million (72.4%) lived in urban areas, 5.8 million (10.3%) in suburban areas, 5.6 million (9.8%) in large rural areas, and 4.2 million (7.5%) in small rural areas (Table 1, Figure 2). This closely aligns with the overall national population distribution by urbanicity (74.1% in urban, 10.5% in suburban, 8.41% in large rural, and 7.01% in small rural areas).

Among 28.9 million individuals who relied on keystone pharmacies for access, 18.8 million (65.2%) lived in urban areas, 3.9 million (13.4%) in suburban areas, 2.1 million (7.1%) in large rural areas, and 4.1 million (14.3%) in small rural areas. Small rural areas represented 7% of the entire US population but 14.3% of the population relying on keystone pharmacy access; suburban areas represented 10.5% of national population but 13.4% of the population relying on keystone pharmacies.

### By State

**Figure 3.**
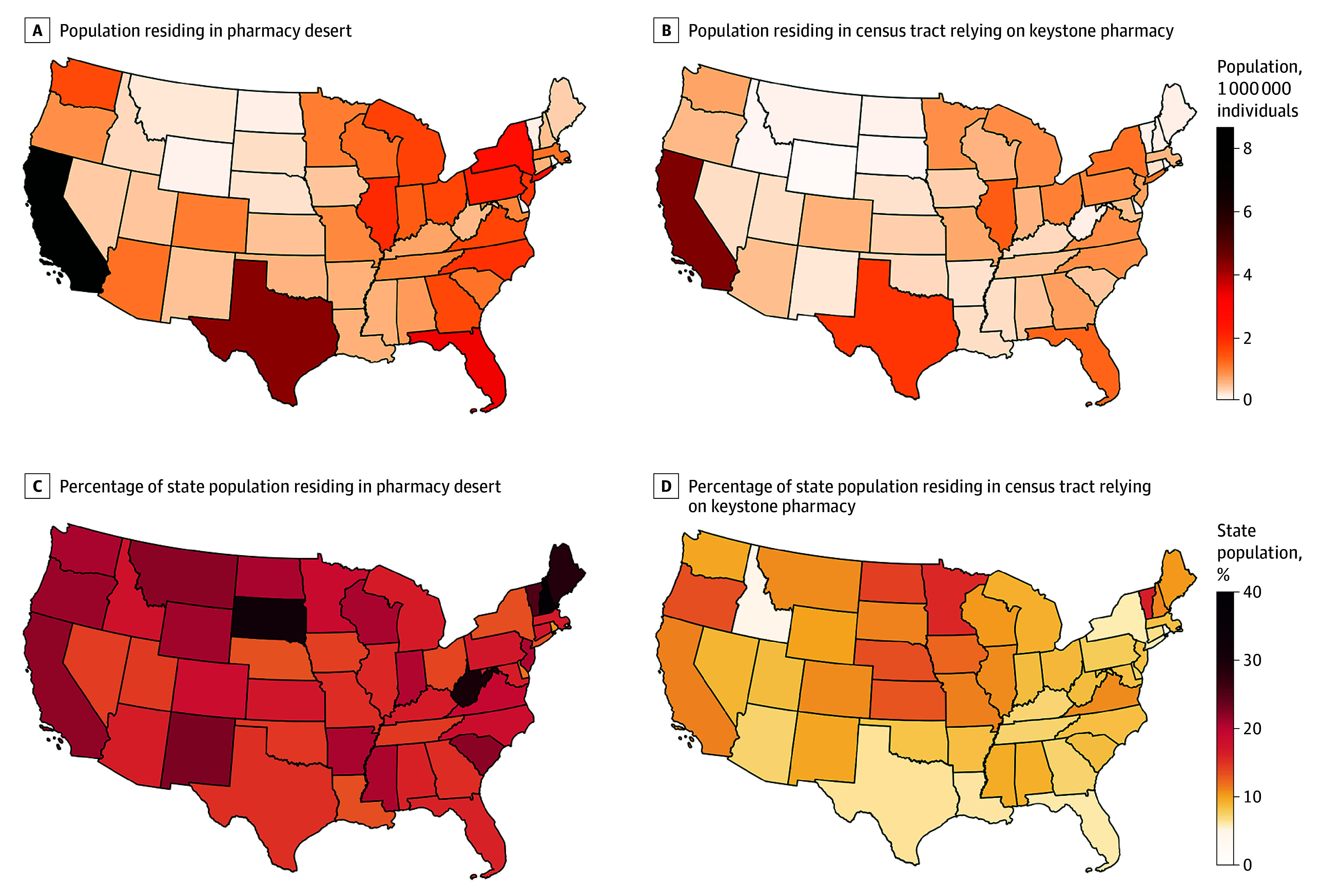
Population by State Residing in Pharmacy Deserts and in Census Tracts Served by Keystone Pharmacies Pharmacy desert was defined as a census tract lacking a pharmacy within the established drive time threshold, based on the region and level of urbanicity. Keystone pharmacy was defined as a critical pharmacy whose removal would result in the conversion of a census tract into a pharmacy desert.

In absolute numbers, California had the largest population living in pharmacy deserts (8.6 million), followed by Texas (4.3 million) and Florida (3.4 million) (Figure 3A; eTable 4 in Supplement 1). Five states had more than 25% of the population residing in pharmacy deserts: New Hampshire (39.3%), South Dakota (31.9%), West Virginia (29.9%), Maine (28.7%), and Vermont (25.3%) (Figure 3C).

In absolute numbers, California was also the state with the largest population relying on keystone pharmacies for access (4.5 million), followed by Texas (1.8 million) and Illinois (1.4 million) (Figure 3B; eTable 4 in Supplement 1). Eighteen states had at least 10% of the population relying on keystone pharmacies for access, including Vermont and Minnesota, which had greater than 15% of the population in census tracts relying on a single pharmacy (Figure 3D).

### By Race and Ethnicity

White individuals were more likely to reside in pharmacy deserts than Black and Hispanic individuals (Table 2). Specifically, White individuals accounted for 66.4% of the US population, but 76.8% of individuals residing in pharmacy deserts. Black and Hispanic individuals accounted for 13.5% and 20.1% of the US population, respectively, but 8.1% and 15.1% of individuals in pharmacy deserts. Similarly, White individuals were more likely to rely on keystone pharmacies for access. Compared with 66.4% of the US population, White individuals represented 72.9% of people relying on keystone pharmacies for access.

**Table 2.** Race Population Totals by Pharmacy Vulnerability Index^a^

Population	Individuals, No. (%)					
	Pharmacy Vulnerability Index					Total (N = 293 762 826)
	0 (n = 52 569 838)	1 (n = 26 273 010)	2-4 (n = 93 618 659)	5-8 (n = 55 686 195)	≥9 (n = 65 615 124)	
Black	4 272 067 (8.1)	2 716 818 (10.3)	12 052 006 (12.9)	8 635 197 (15.5)	12 102 426 (18.4)	39 778 514 (13.5)
Hispanic	7 934 372 (15.1)	4 399 322 (16.7)	18 325 932 (19.6)	11 918 170 (21.4)	16 418 698 (25.0)	58 996 494 (20.1)
White	40 363 399 (76.8)	19 156 870 (72.9)	63 240 721 (67.6)	35 132 828 (63.1)	37 094 000 (56.5)	194 987 818 (66.4)

^a^ Totals reflect summation of only these 3 racial and ethnic groups. Hence, overall total is less than national population listed in Table 1.

Of note, though most (72.9%) of the US population reside in urban settings, Black and Hispanic individuals are overrepresented in urban settings (84.5% and 85.7%, respectively) while nonurban RUCA classes were overrepresented by White individuals (eTable 5 in Supplement 1).

### Top Keystone Pharmacies by Population Served

eTable 6 in Supplement 1 lists the location of the top 10 keystone pharmacies ranked by population relying on them for access. For example, an independent pharmacy in Oxnard, California, was the keystone pharmacy for 7 census tracts, with as many as 34 346 individuals relying on this pharmacy for access.

## Discussion

We deployed a novel method to identify areas with suboptimal pharmacy access (pharmacy deserts) through comparison with driving times to supermarkets and while accounting for differences in normative drive times across regions and urbanicity levels. Our analysis identified that 57.1 million individuals, or 17.7% of the US population, resides in pharmacy deserts, with an additional 28.9 million individuals or 8.9% of the US population relying on a single pharmacy (keystone pharmacy) for access. Notably, independently owned pharmacies were more likely than chain pharmacies to be keystone pharmacies. The most rural settings were disproportionately reliant on keystone pharmacies for access.

Our methodology to measure pharmacy accessibility introduced 2 novel aspects. The first is a travel time threshold to define access based on empirical data, which adjusts according to the region and level of urbanization. This approach has important advantages over conventional approaches: (1) it accounts for a nuanced spectrum of urbanicity levels beyond the binary urban-rural classification; (2) it uses drive time thresholds that are derived empirically instead of a priori convenience cutoffs; and (3) thresholds reflect urbanicity and regional norms.^21^ The second innovation of our methodology—the measurement of a vulnerability index—enables a nuanced understanding of pharmacy accessibility, distinguishing not only between desert and nondesert areas but also identifying regions reliant on a single pharmacy location for access.

This approach is critical to informing policymaking, as it allows for the identification of areas at risk of becoming deserts. These areas can serve as targets of policy interventions designed to prevent an exacerbation of inequities in health care access. To support pharmacy access, states could implement incremental dispensing fees or reimbursement rates for Medicaid prescriptions filled by keystone pharmacies to foster their financial sustainability.^29^ We find analogous examples in food policy where governmental programs at the city,^30^ state,^31^ and federal^32^ levels demonstrate how zoning incentives, tax breaks, and direct loans can be used to attract and retain supermarkets in underserved areas.

Our analyses provide important insights into national variation in pharmacy access, beyond the uneven distribution of keystone pharmacies across the rural-urban continuum noted. First, while the most populous states have the highest absolute numbers of people living in pharmacy deserts and relying on keystone pharmacies, less populous states such as New Hampshire, South Dakota, West Virginia, Maine, and Vermont have disproportionately high percentages of their populations affected. This highlights the widespread nature of pharmacy access issues across both densely and sparsely populated states. Second, independently owned pharmacies were more likely to be keystone pharmacies than chain pharmacies. This observation demonstrates the relevance of independent pharmacy sustainability in the prevention of an exacerbation in inequities in pharmacy access across the country. The financial viability of independent pharmacies has been threatened by the increasing vertical and horizontal consolidation of pharmacy benefit managers in recent years, which has led to the exclusion of independent pharmacies from the preferred pharmacy networks of major insurers.^33^ The reform of the business practices of pharmacy benefit managers has been one of the active targets of health care reform; for example, recent legislation aimed to mandate Part D plans to include in preferred pharmacy networks 80% of independent pharmacies, compared with 50% of not independently owned pharmacies.^34^

Some may argue that the increased popularity of mail-order pharmacies may partially mitigate pharmacy access challenges. Beyond medication dispensing, pharmacies provide many valuable services that require direct patient interaction, such as the provision of vaccinations; these services cannot be replaced through online or mail-order pharmacies. Moreover, the use of mail-order pharmacies is disproportionately higher among non-Hispanic White, higher-income, college-educated, and privately insured individuals, suggesting that disparities in adoption may limit their overall impact, particularly for disadvantaged populations.^35^

The methodology demonstrated in this first report using pharmacies as a case study can be easily extended to measuring access to other critical health care facilities, such as hospitals and emergency departments. Such applications could support the development of interventions that ensure the financial sustainability of facilities serving as the single source of hospital or emergency department services in an area.

Using supermarket access as a model for setting regional and urbanicity-specific drive time thresholds for pharmacy access is both a precedent and practical choice. Initially, the concept of food deserts relied on supermarket access metrics, which have successfully been applied to identifying pharmacy deserts in previous studies.^8,9^ Supermarkets are almost universally needed and used, making them a reasonable reference for gauging market supply responses. Their common presence helps ensure that drive time thresholds based on supermarkets are realistic and regionally pertinent, thereby improving the accuracy and applicability of pharmacy access analysis.

### Limitations

We acknowledge that our study has limitations, including the reliance on comparisons with supermarket access. Our method presumes that supermarkets are optimally distributed to meet demand and that the market demand for pharmacies mirrors that for supermarkets. In other words, if used uncritically, our reliance on supermarket coverage as a baseline could overlook or even exacerbate pharmacy access challenges in communities already facing inadequate food resources, underscoring the need for careful, context-specific application of these findings and further analyses targeting at-risk populations.

Additional limitations should be considered in the interpretation of our findings. First, our approach assumes that individuals choose pharmacies based solely on minimizing distance or drive time from their homes, which may not accurately reflect real-world behavior.^36^ Second, our analyses relied on driving time as a measure of travel time, which assumes access to a personal vehicle and may not capture the experiences of individuals relying on public transportation. Additionally, future work should evaluate the role of racial and ethnic distribution across the rural-urban continuum in explaining the disparities in pharmacy access noted across racial and ethnic groups.

## Conclusions

Our study uses a novel analytic approach to highlight substantial differences in pharmacy access across the United States, with 17.7% of the population residing in pharmacy deserts and 8.9% relying on a single pharmacy for access. Residents of small rural areas were particularly at risk, with a higher dependency on keystone pharmacies for access. The methods used in this analysis, including regionally adapted travel time thresholds and a vulnerability index algorithm, provide a nuanced understanding of pharmacy accessibility that can be applied to the measurement of access to other critical resources. Through the identification of areas that rely on a single pharmacy for access, our research can support the implementation of targeted policy efforts to prevent further exacerbation of inequities in pharmacy access.

## Notes

Supplement 1. **eFigure.** Map of Commuter Regions From Nelson & Ray (2016) **eTable 1.** Table of Travel Time Thresholds by Region and RUCA Class Derived From Supermarket Analysis **eTable 2. **Table Mapping RUCA Secondary Codes to Urban, Suburban, Large Rural, and Small Rural Classes **eTable 3.** Dispenser Class Distribution of Unique Keystone Pharmacies **eTable 4.** State Population in Desert and Keystone Census Tracts **eTable 5.** Population by Race and RUCA Class **eTable 6.** Top Ten Keystone Pharmacies by Number of Individuals Relying on Them for Access

Supplement 2.
Data Sharing Statement
