# Error in Author Affiliation

**DOI:** 10.1001/jamanetworkopen.2025.11341

**Published:** 2025-04-15

In the Original Investigation titled “Vulnerability Index Approach to Identify Pharmacy Deserts and Keystone Pharmacies,”^1^ published March 13, 2025, there was an error in an author affiliation in the byline. Dr Kahn was incorrectly listed as being affiliated with Sanofi. Dr Kahn was not affiliated with Sanofi during the conduct of this study. This article has been corrected.^1^
