# Supplementary material for: Vulnerability Index Approach to Identify Pharmacy Deserts and Keystone Pharmacies
Source: JAMA Netw Open. 2025 Mar 13;8(3):e250715. doi: 10.1001/jamanetworkopen.2025.0715 (PMC11907307; doi:10.1001/jamanetworkopen.2025.0715)
Supplement: Supplement 1. — eFigure. Map of Commuter Regions From Nelson & Ray (2016) eTable 1. Table of Travel Time Thresholds by Region and RUCA Class Derived From Supermarket Analysis eTable 2. Table Mapping RUCA Secondary Codes to Urban, Suburban, Large Rural, and Small Rural Classes eTable 3. Dispenser Class Distribution of Unique Keystone Pharmacies eTable 4. State Population in Desert and Keystone Census Tracts eTable 5. Population by Race and RUCA Class eTable 6. Top Ten Keystone Pharmacies by Number of Individuals Relying on Them for Access [file jamanetwopen-e250715-s001.pdf]

Supplemental Online Content

Mathis WS, Berenbrok LA, Kahn PA, Appolon G, Tang S, Hernandez I. Vulnerability index approach to identify pharmacy deserts and keystone pharmacies. *JAMA Netw Open.* 2025;8(3):e250715. doi:10.1001/jamanetworkopen.2025.0715

eFigure. Map of Commuter Regions From Nelson & Ray (2016)

eTable 1. Table of Travel Time Thresholds by Region and RUCA Class Derived From Supermarket Analysis

eTable 2. Table Mapping RUCA Secondary Codes to Urban, Suburban, Large Rural, and Small Rural Classes

eTable 3. Dispenser Class Distribution of Unique Keystone Pharmacies

eTable 4. State Population in Desert and Keystone Census Tracts

eTable 5. Population by Race and RUCA Class

eTable 6. Top Ten Keystone Pharmacies by Number of Individuals Relying on Them for Access

This supplemental material has been provided by the authors to give readers additional information about their work.

© 2025 Mathis WS et al. *JAMA Network Open*.

eFigure. Map of commuter regions from Nelson & Ray (2016).[21]

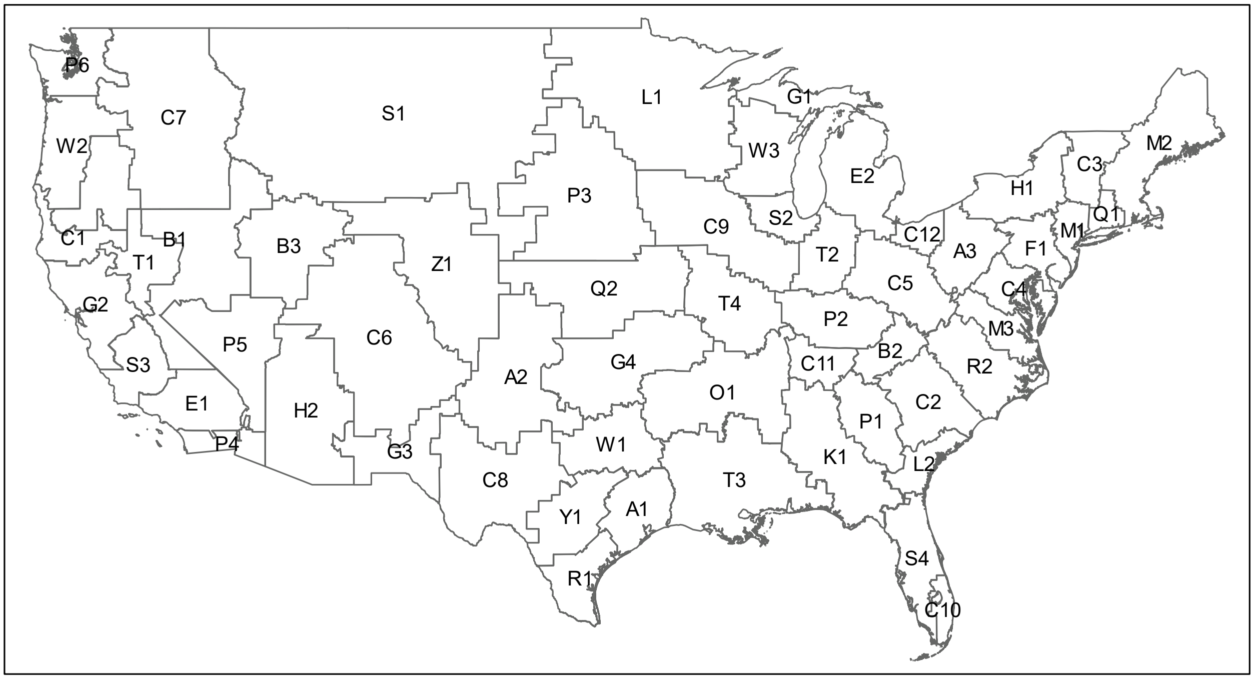

© 2025 Mathis WS et al. *JAMA Network Open*.

eTable 1. Table of travel time thresholds by region and RUCA class derived from supermarket analysis

	Urban	Suburban	Large Rural	Small Rural		Urban	Suburban	Large Rural	Small Rural
**A1**	4.84	16.7	14.44	19.13	**L2**	6.36	17.89	14.35	16.34
**A2**	4.72	17.85	5.66	19.77	**M1**	3.33	13.03	11.12	13.87
**A3**	6.78	17.14	13.3	16.53	**M2**	5.83	15.3	14.58	16.78
**B1**	5.47	18.1	10.83	20.37	**M3**	5.18	18.55	11.46	18.46
**B2**	8.22	18.32	16.26	16.61	**O1**	5.78	18.24	14.82	18.77
**B3**	4.74	13.98	7.24	16.22	**P1**	6.49	17.12	13.32	17.38
**C1**	5.56	18.63	8.34	29.01	**P2**	5.53	18.43	14.44	17.92
**C2**	6.85	16.03	13.23	16.63	**P3**	4.6	18.54	10.05	20.4
**C3**	5.96	17.12	14.3	17.37	**P4**	4.4	16.1	4.32	43.08
**C4**	5	15.5	10.62	12.99	**P5**	4.39	24.23	12.19	54.62
**C5**	6.03	17.41	15.54	18.18	**P6**	5.14	14.95	12.56	19.42
**C6**	5.16	17.97	13.54	32.05	**Q1**	5.77	13.99	7.87	17.34
**C7**	5.27	16.34	11.22	24.23	**Q2**	5	16.04	12.1	20.93
**C8**	6.22	22.19	7.61	15.17	**R1**	5.46	18.4	8.18	16.11
**C9**	5.21	19.43	13.56	18.44	**R2**	5.99	16.2	12.01	17.48
**C10**	4.15	17.21	8.99	18.69	**S1**	6.98	21.78	9.47	25.46
**C11**	7.04	16.67	16.44	18.65	**S2**	4.16	11.33	10.05	16.26
**C12**	5.1	14.16	10.44	13.3	**S3**	4.22	12.85	4.44	17.66
**E1**	3.34	8.12	16.13	16.65	**S4**	6.15	15.93	14.04	19.98
**E2**	5.1	15.98	13.59	17.44	**T1**	6.31	15.25	10.54	32.03
**F1**	5.21	14.34	9.75	14.02	**T2**	6.12	18.65	13.1	15.75
**G1**	NA	NA	14.38	21.25	**T3**	6.51	19.22	16.46	20.51
**G2**	3.84	13.18	8.98	15.11	**T4**	5.9	19.21	15.18	18.29
**G3**	5.05	16.44	12.23	25.16	**W1**	4.68	15.92	14.16	17.92
**G4**	5.6	18.58	14.23	18.81	**W2**	4.41	14.01	8.15	18.93
**H1**	5.41	15.35	10.68	17.14	**W3**	5.29	15.44	12	17.18
**H2**	4.51	14.81	14.75	29.55	**Y1**	6.09	18.63	13.2	19.9
**K1**	6.81	19.18	14.2	17.37	**Z1**	4.72	20.73	9.33	19.71
**L1**	5.23	15.78	14.08	21.37					

© 2025 Mathis WS et al. *JAMA Network Open*.

eTable 2. Table mapping RUCA secondary codes to urban, suburban, large rural, and small rural classes.

Level	Secondary RUCA Codes
Urban core	[1.0, 1.1]
Suburban	[2.0, 2.1, 3.0]
Large rural	[4.0, 4.1, 4.2, 5.0, 5.1, 5.2, 6.0, 6.1]
Small town/rural	[7.0, 7.1, 7.2, 7.3, 7.4, 8.0, 8.1, 8.2, 8.3, 8.4, 9.0, 9.1, 9.2,10.0, 10.1, 10.2, 10.3, 10.4, 10.5, 10.6]

Scheme 1 from Hailu & Wasserman, 2016.[19]

Urban Core: contiguous built-up areas of 50,000 people or more. These areas correspond to the US Census Bureau’s urbanized areas.

Suburban: areas, often in metropolitan counties, with primary high commuting flows to urban cores (e.g., Eatonville in Pierce County) and all other areas with secondary commuting flows of 30%-49% of the population to urban cores.

Large Town: towns with populations of 10,000-49,999 and surrounding rural areas with 10% or more primary commuting flows to these towns, and towns with secondary commuting flows of 10% or more to Urban Cores.

Small Town/Rural Areas: towns with populations below 10,000 and surrounding commuter areas with more than a one-hour driving distance to the closest city.

© 2025 Mathis WS et al. *JAMA Network Open*.

eTable 3: Dispenser Class Distribution of Unique Keystone Pharmacies

Dispenser Class	Keystones		Total	
Chain	3,047	(54.3%)	36,456	(59.7%)
Independent or Franchise	2,519	(44.9%)	24,288	(39.8%)
Government	44	(0.8%)	302	(0.5%)
Total	5,610	(100%)	61,046	(100%)

Chain pharmacies include pharmacy locations that are part of a group of four or more pharmacies under common ownership.

Independent pharmacies and franchise pharmacies were bundled together as they both represent independently owned pharmacies, with or without franchise agreements.[27]

Government pharmacies include those under the jurisdiction of federal, state, county or city government or the Indian Health Service.[28]

© 2025 Mathis WS et al. *JAMA Network Open*.

eTable 4: State Population in Desert and Keystone Census Tracts.

State Name	Total Population	Population in Pharmacy Deserts		Population in Census Tracts Relying on Keystone Pharmacies	
		n	%	n	%
Alabama	4,892,115	789,381	16.14%	463,102	9.47%
Arizona	7,148,191	1,180,510	16.51%	534,554	7.48%
Arkansas	3,011,391	625,875	20.78%	257,171	8.54%
California	39,234,027	8,642,203	22.03%	4,454,056	11.35%
Colorado	5,668,232	1,046,801	18.47%	617,266	10.89%
Connecticut	3,565,371	617,542	17.32%	242,548	6.80%
Delaware	967,416	115,721	11.96%	72,771	7.52%
District of Columbia	695,846	0	0.00%	0	0.00%
Florida	21,180,888	3,407,281	16.09%	1,302,962	6.15%
Georgia	10,511,338	1,617,170	15.39%	775,122	7.37%
Idaho	1,754,367	310,942	17.72%	86,116	4.91%
Illinois	12,715,579	1,995,652	15.69%	1,375,466	10.82%
Indiana	6,684,409	1,369,272	20.48%	580,421	8.68%
Iowa	3,148,731	449,930	14.29%	390,267	12.39%
Kansas	2,912,009	500,772	17.20%	382,929	13.15%
Kentucky	4,456,085	741,741	16.65%	326,305	7.32%
Louisiana	4,661,796	627,203	13.45%	297,135	6.37%
Maine	1,340,825	385,127	28.72%	139,985	10.44%
Maryland	6,023,087	991,893	16.47%	509,478	8.46%
Massachusetts	6,862,913	1,153,462	16.81%	586,195	8.54%
Michigan	9,938,869	1,656,352	16.67%	923,189	9.29%
Minnesota	5,598,276	1,055,019	18.85%	882,265	15.76%
Mississippi	2,981,759	617,221	20.70%	288,138	9.66%
Missouri	6,124,160	947,135	15.47%	693,749	11.33%
Montana	1,061,178	234,654	22.11%	114,612	10.80%
Nebraska	1,922,373	257,850	13.41%	260,712	13.56%
Nevada	3,029,538	438,696	14.48%	276,408	9.12%
New Hampshire	1,355,244	532,993	39.33%	150,916	11.14%
New Jersey	8,873,054	1,830,216	20.63%	757,015	8.53%
New Mexico	2,094,258	475,521	22.71%	203,320	9.71%
New York	19,448,146	2,622,452	13.48%	1,180,554	6.07%
North Carolina	10,377,099	1,901,803	18.33%	883,437	8.51%
North Dakota	760,394	158,450	20.84%	108,176	14.23%
Ohio	11,665,072	1,638,334	14.04%	1,030,037	8.83%

© 2025 Mathis WS et al. *JAMA Network Open*.

eTable 4 (continued)

State Name	Total Population	Population in Pharmacy Deserts		Population in Census Tracts Relying on Keystone Pharmacies	
		n	%	n	%
Oklahoma	3,946,617	580,129	14.70%	327,182	8.29%
Oregon	4,176,346	891,972	21.36%	567,106	13.58%
Pennsylvania	12,765,612	2,167,528	16.98%	1,009,611	7.91%
Rhode Island	1,057,798	112,940	10.68%	55,518	5.25%
South Carolina	5,069,928	1,124,430	22.18%	443,536	8.75%
South Dakota	879,336	280,789	31.93%	98,317	11.18%
Tennessee	6,765,351	989,445	14.63%	505,409	7.47%
Texas	28,571,668	4,345,165	15.21%	1,848,555	6.47%
Utah	3,148,958	462,097	14.67%	274,025	8.70%
Vermont	624,340	157,745	25.27%	102,406	16.40%
Virginia	8,491,738	1,643,744	19.36%	927,764	10.93%
Washington	7,483,162	1,566,743	20.94%	732,268	9.79%
West Virginia	1,807,426	540,254	29.89%	156,894	8.68%
Wisconsin	5,805,738	1,212,857	20.89%	602,406	10.38%
Wyoming	581,348	123,362	21.22%	58,855	10.12%

© 2025 Mathis WS et al. *JAMA Network Open*.

eTable 5: Population by Race and RUCA class

RUCA Class	Black	Hispanic	White	Total
SmallRural	1,568,617 (3.9%)	1,889,636 (3.2%)	18,027,405 (9.2%)	21,485,658 (7.3%)
LargeRural	2,320,215 (5.8%)	3,234,016 (5.5%)	20,329,048 (10.4%)	25,883,279 (8.8%)
Suburban	2,295,288 (5.8%)	3,341,135 (5.7%)	26,648,781 (13.6%)	32,285,204 (11.0%)
Urban	33,609,664 (84.5%)	50,567,485 (85.7%)	130,251,261 (66.7%)	214,428,410 (72.9%)
Total	39,793,784 (100%)	59,032,272 (100%)	195,256,495 (100%)	294,082,551 (100%)

Note: Totals reflect summation of only these three racial/ethnic groups. Hence overall total is less than national population listed in Table 1.

© 2025 Mathis WS et al. *JAMA Network Open*.

eTable 6: Top Ten Keystone Pharmacies by Number of Individuals Relying on Them for Access.

Population	Number of Census Tracts Served	City	State	Dispenser Class
34,346	7	Oxnard	CA	Independent
30,680	6	Columbia	SC	Chain
26,387	6	Tucson	AZ	Chain
25,093	5	Los Angeles	CA	Independent
24,540	4	Portland	OR	Chain
24,245	3	Raleigh	NC	Chain
24,102	4	San Diego	CA	Chain
23,763	5	Bakersfield	CA	Chain
23,604	5	Tracy	CA	Chain
23,084	5	Sacramento	CA	Chain

© 2025 Mathis WS et al. *JAMA Network Open*.
