# Supplementary material for: Vulnerability Index Approach to Identify Pharmacy Deserts and Keystone Pharmacies
Source: JAMA Netw Open. 2025 Mar 13;8(3):e250715. doi: 10.1001/jamanetworkopen.2025.0715 (PMC11907307; doi:10.1001/jamanetworkopen.2025.0715)
Supplement: Supplement 2. — Data Sharing Statement [file jamanetwopen-e250715-s002.pdf]

Data Sharing Statement

Mathis. Vulnerability Index Approach to Identify Pharmacy Deserts and Keystone Pharmacies. *JAMA Netw Open*. Published March 13, 2025. doi:10.1001/jamanetworkopen.2025.0715

Data
Data available: No

Additional Information
Explanation for why data not available: The fundamental data set used in this analysis -- pharmacy data -- was proprietary and cannot be shared per the licensing of the database vendor.
